# The Need for Management Capacity to Achieve VISION 2020 in Sub-Saharan Africa

**DOI:** 10.1371/journal.pmed.1000184

**Published:** 2009-12-08

**Authors:** Susan Lewallen, Amir Bedri Kello

**Affiliations:** 1 Kilimanjaro Centre for Community Ophthalmology, Good Samaritan Foundation, Moshi, Tanzania; 2 Light for the World, Addis Ababa, Ethiopia

## Abstract

Susan Lewallen and Amir Bedri Kello argue that human resources management will be crucial in reaching the global goal of eliminating avoidable blindness by the year 2020.

**Summary Points**

- Sub-Saharan Africa has the highest regional burden of blindness and visual impairment in the world.
- More clinically trained manpower alone will not be sufficient to meet eye care needs and will not guarantee productive services.
- Management systems run by professional nonclinical personnel are needed to support clinical personnel so that they can be productive.
- There is currently a lack of such systems and personnel and without a change, VISION 2020 goals in Africa will not be realized.

Globally, there are 314 million people with vision impairment, of whom 269 million people have low vision and 45 million are blind [1],[2]. The geographic distribution of visual impairment is not uniform and more than 90% of the world's visually impaired live in developing countries. Sub-Saharan Africa, with around 11% of the world's population, has about 20% of the world's blindness [1]. This represents the highest regional burden of blindness in the world. VISION 2020: The Right to Sight is a broad initiative whose goal is to eliminate avoidable blindness by the year 2020 (see Box 1). Whether this initiative goes the way of other unrealized slogans of the past, or whether it can actually be met still remains to be seen. In sub-Saharan Africa, there are still vast populations with limited or no access to eye services. Progress has been made in some places, however, and there are lessons we can learn from these that will help us come closer to realizing VISION 2020 goals. In this article, we examine the need for dedicated managers if VISION 2020 goals are to be achieved in Africa.

**Box 1. The VISION 2020 Initiative**

“VISION 2020: The Right to Sight” is an initiative aimed at eliminating avoidable blindness by the year 2020. It was launched by the World Health Organization and the International Agency for the Prevention of Blindness (a group of international NGOs, professional associations, eye care institutions, academic institutions, and corporations) in 1999. The aim is reasonable in theory, since practical, cost-effective strategies already exist for the major causes of avoidable vision loss: cataract, refractive errors, trachoma, onchocerciasis, and childhood blindness. The initiative encourages overall national planning but emphasizes the need for implementation at the administrative level of approximately 1,000,000 population. Cooperation rather than competition among governmental and nongovernmental partners is encouraged to make maximum use of existing resources.

## The Lack of Comprehensive Eye Services

Inadequate human resources have long been noted among the constraints to better eye health care [3]; the ophthalmologist-to-population ratio for much of sub-Saharan Africa countries is usually accepted to be around 1∶1,000,000 [4]. Therefore, one of the three prongs of the VISION 2020 strategy is human resource development, including the training of more personnel. This goal usually seems to be interpreted as a need for more personnel with clinical training. While the inadequate numbers of doctors and nurses providing eye care in Africa is indisputable, sheer numbers, either trained or working, should not be the main indicators of progress. What happens after training? Is the training applied effectively? How productive are workers and what factors influence this?

Although there is increasing awareness throughout Africa of the complex but interrelated problems in human resources for health, including attrition, absenteeism, and productivity [5],[6], there is little specifically related to eye health workers. One study of cataract surgeons (a mid-level cadre) demonstrated that, on average, these surgeons performed fewer than 250 cataract surgeries per year with a median of 113 [7]. This number is very low, considering that even operating on ten cases twice a week (not a heavy burden) for 40 weeks would result in 800 surgeries in a year. Interestingly the variation in productivity was extreme, ranging from less than 50 to 750 surgeries per year. The factors significantly associated with higher productivity included adequate equipment (microscope and surgical instruments), enough support staff, and a supporting outreach program that provided transportation for patients to get to the hospital [7]. The study did not attempt to measure all of the critical but more subjective factors that are associated with productivity, such as leadership. A study of trichiasis surgeons (another mid-level cadre) found again that productivity was associated with having an outreach programme and having adequate equipment and, in this study, knowing who one's supervisor was [8].

The VISION 2020 initiative advocates for planning for comprehensive eye services at the level of 1–2 million people—referred to as a “district” (although populations of 1–2 million are often comprised in a “Region” or “Province”). This population-based approach is a change from the old model, which was based on services provided at a given hospital, regardless of where patients came from. The new model calls for much more coordination and linkages of services among all the eye care providers and donors to eye care in a district, whether they be government, private, or mission based. Our experience in observing and mentoring programmes as they plan and implement VISION 2020 programmes has led us to recognize that successful programs include a specific management component, consisting of management systems and skills in the broadest sense while less productive programs lacked this component. We divide this management component into several categories for discussion, recognizing that management issues cut across many areas.

### Human Resources Management

Eye care, like much medical care, must be delivered by a team effort. The best trained ophthalmologist still needs a team to be effective. An eye care team usually comprises the ophthalmologist, ophthalmic nurses (a role sometimes served by ophthalmic technicians or ophthalmic assistants depending on the country), refractionist or optometrist, and other support staff (cleaners, security personnel, and drivers). However, ideally it should also include an eye care manager, patient counselor, equipment and instrument maintenance technician, and community eye care coordinator. The roles and responsibilities of these team members have been described [9]. Teamwork requires management of people, with every team member knowing his or her job, showing up to work, and performing satisfactorily [10]. Management at this level involves a wide range of issues including skills development, routine monitoring of output, assessment of the quality of outcomes, supportive supervision, conflict resolution, and effective systems for performance appraisal. Workers must know what their tasks are and when they are performing well and receive help when they are not. When a worker is absent, someone needs to be deputized to take his place. Team workers need motivation, not just by increased salaries, which is often temporary [11], but by an organizational culture built on shared values and maintained through good leadership [12]. This latter should come from the clinical head of a service, but he or she can get tremendous support from a well-trained nonclinical manager who is empowered to deal with many details. It is unrealistic to expect the clinical head to provide all the human resource management needed.

### Financial and Materials Management

Provision of eye care services requires a reliable supply of special medicines and other consumables, many of which are not stocked by government medical stores. Eye care, particularly cataract surgery, has changed dramatically in the past 15–20 years in Africa. Microscopic surgery with an intraocular lens is the standard of care now. Subspecialists in paediatric ophthalmology and retinal surgery are being trained to meet the needs of the people. Making use of these highly trained doctors requires both basic and some rather sophisticated, often expensive equipment, which must be procured and usually repaired outside the country and requires special attention to maintenance to get maximum usage. How do we prevent the all-too-often-encountered scenario in which the surgeon finds out that his supply of sutures finished “yesterday” or the equipment is simply unusable for lack of a spare bulb?

Eye care in poor countries has traditionally been supported by charities or donors with a special interest in blindness. As a result of this and competing priorities, most governments provide little support for eye care, often limited to the salaries of a few workers. To provide proper services for patients, someone usually has to seek funds from external donors. Sometimes this is a motivated doctor, eager to use his training, or perhaps a mission organization eager to include eye care amongst its services. External donors are essential in providing eye care in sub-Saharan Africa, but, understandably, they demand considerable reporting and accountability with funds. These tasks are time consuming. Who will seek the necessary external funding, see that it is used well, and account for it?

### Management Information

Good planning, whether at the global, regional, national, or district level, requires accurate information. This accuracy is especially important since VISION 2020 planning is focused on the needs of a specific population and not just a hospital. Whether information comes from sleek computer systems or from crumbling, dog-eared “counter books,” it must be collected properly and analysed if it is to be meaningful. It must be passed upwards in a timely fashion to be pooled and compared with other information at a national or global level. Ideally, feedback comes back down the information ladder too, where it is used to help workers understand their work and improve their performances. Collecting meaningful data and reporting it regularly takes time and requires systems that are understood and used by the workers consistently. Who does this for an eye service?

### Outreach Services

In providing eye services for the elderly in Africa (in whom 80% of all visual disability occurs), it is well known that patients do not make their way to the hospital without considerable help [13]–[15]. They face many barriers in accessing services at a hospital and some “bridge” must be built between the hospital and the communities to allow access. Indeed, this is a key concept in the VISION 2020 strategy. Simply referring patients from peripheral health centres and expecting them to make their own way to a hospital where quality eye surgery is offered does not work. Sending operating teams out to rural, inadequately equipped settings to run “camps” is not recommended [16]. Many programmes serving rural populations have to offer transportation to get these elderly people to the hospital for high quality cataract surgery [17]–[19]. Whatever strategy is agreed upon, it will require planning, implementation, ongoing supervision, and monitoring. Without these bridging programmes to link patients with hospitals, we know that eye services will be underutilized while patients remain visually impaired. Who will organize and run these programs?

A model, well-functioning VISION 2020 program would ensure that a population of 1–2 million will all have access to eye services. Programmes in every “district” in Africa would allow us to eliminate most avoidable blindness. Each programme would include a hospital service at which reasonably high level care was available, including refractive services, adult cataract surgery, maybe glaucoma surgery, emergency treatment for minor/moderate trauma, and medical treatment for many infectious and inflammatory eye conditions. Some complicated cases would require referral to a tertiary care facility.

There would be some “bridging strategy” out to the communities where most elderly visually impaired people live. The district would send regular reports of the conditions they treat to the national level, where the information would be aggregated and used for national planning purposes. The hospital at the “centre” of a VISION 2020 plan might be run by a mission, especially where there are already functioning eye units, or it might be a government hospital with support from external donors. Whichever, it will clearly require a high degree of organization and management to make the program run smoothly. Among several well functioning VISION 2020 programmes described in the literature, which do provide services to rural people [17]–[19], some are government and some private or mission. One feature all have in common is a nonclinical, designated manager to support the program and clinicians who work with the manager.

## Our Proposal

Ideally a hospital that was central to a VISION 2020 program serving 1–2 million people would ensure that there was a manager to work alongside the surgeon head, so that things ran well within the hospital and an outreach service was organized (see Box 2). Indeed, at purely private (mission) hospitals, at least those that are productive, this situation is often in place. But most ministries of health have limited health system managers to spare and often are not willing to provide those they have to eye programs.

**Box 2. Key Managerial Issues in a District VISION 2020 Programme**

- Good human resource management
  - Appropriate skills development
  - Supportive supervision at all levels (including performance appraisal, conflict resolution, accountability, motivation)
- Improved financial and materials management
  - Reliable purchasing and accurate inventory
  - Routine equipment maintenance
  - Timely and accurate financial reporting
- Outreach program logistics to link community with hospital

We suggest that several things can be done to address the problem For the foreseeable future, nongovernmental organisations (NGOs) will have to support eye care programs at ministry of health facilities if we are to meet VISION 2020 goals. Structuring the eye service, both at the hospital and the outreach level, as a program, and providing an effective manager specifically for the program is one step towards accomplishing these goals. Of course, in each situation, the ministry of health and the NGO will have to reach an understanding of how the manager is paid and to whom he is responsible; both may have to be flexible. One of the latest buzz phrases in health care development is “health systems strengthening,” a phrase that means different things to different people and organizations [20]. Surely, however, strengthening human resource, materials, and financial management systems and ensuring there are managers to run these ought to be included.

It is sometimes argued that it isn't “fair” for eye departments to have managers when other specialty departments don't. It is likely that many should, particularly if they provide outreach to communities. We view this as an opportunity for eye departments to lead the way in experimenting with models and showing what can be done. There are a number of specialist services that cannot realistically be included in primary health care. Although we have used the VISION 2020 initiative, concerned with prevention of blindness as our example here, we believe that developing a cadre of good health care managers is essential for health care provision in all areas of medicine including both clinical and public health services.

At the same time, ophthalmologists (and other doctors) need to learn more about basic management themselves, so that they can work alongside managers instead of compete with them for perceived power. Leadership can come from the doctors, but managers must be empowered to be effective.

Current residency training programmes concentrate on clinical knowledge and skills, leaving ophthalmologists ill-equipped to deal with the challenges of leading and managing eye care units, which they are often expected to do. We have often encountered ophthalmologists running departments who were unaware of the concept of participatory performance appraisal or supportive supervision, and how these could improve motivation. These knowledge gaps should be rectified. Basic up-to-date management principles should be included in the curriculum as part of basic public health training.

Meanwhile, ministries of health need to recognize the critical role of management in running an eye care department and outreach services. They need to plan for developing such a cadre to support doctors who head departments.

Managerial support would free up ophthalmologists to concentrate on doing what they do best and generally prefer to do. Without recognition of the critical role of management in delivering eye care, even hundreds of newly trained ophthalmologists will not be sufficient to achieve VISION 2020 goals in sub-Saharan Africa.
